# Supplementary material for: Service user experiences of community services for complex emotional needs: A qualitative thematic synthesis
Source: PLoS One. 2021 Apr 29;16(4):e0248316. doi: 10.1371/journal.pone.0248316 (PMC8084224; doi:10.1371/journal.pone.0248316)
Supplement: S1 File — (PDF) [file pone.0248316.s002.pdf]

S1 File

Contents

1 – Search strategy: conceptual purpose 3
2 – Methods 9
3 – Extended Results 10
1 - The long treatment journey 10
1.1 Skills learnt in treatment 10
1.2 Benefits and challenges of treatment 10
1.3 Change over time 11
1.4 Meaning 11
2 - Client-clinician relationship 12
2.1 - Positive qualities for clinicians and the therapeutic relationship 12
2.3 - Relationship dynamics and structure 14
2.4 - Control: involvement in care and choosing who their clinicians are 14
3) Mental Health Services 14
3.1 - Access to services 14
3.2 Experiences of Mental Health Services 15
3.3 - Continuity of care 16
4 – Peer relationships 17
4.1 Family and friends 17
4.2 Peer support 17
4.3 Group treatment 18
5 - Engaging with treatment: psychological barriers and facilitators 19
5.1 - Barriers to treatment 19
5.2 - Facilitators of treatment 19
5.3 - Recovery 20
6 – Components of treatment 20
6.1 - Individualised treatment 20
6.2 - Structured Approach 21
6.3 - Self-Harm 21
6.4 - Medications 22
7 – Diagnosis 23

7.1 Self-understanding 23
7.2 Access to treatment and communication of diagnosis 24
References 25

1 – Search strategy: conceptual purpose

The purpose of this search is to identify studies reporting provision of community-based care for people with diagnosed personality disorder. The search strategy was developed by the review team and it incorporates feedback from our PPI group (the LEWG) who worked with us on this review.

The bibliographic search strategy set out below aims to identify published studies or reports. This search narrative seeks to illustrate the conceptual purpose of the search strategy and the contextual detail of the search syntax reported below (Cooper et al. 2018). Separate search strategies – based on the syntax reported below – will be devised for web-searching.

#	Searches	Results	
1	exp *Personality Disorders/	28263	1. This controlled indexing term has been exploded (indicated by exp) to capture other relevant MeSH indexing terms, such as:<br>• Antisocial Personality Disorder/<br>• Borderline Personality Disorder/<br>• Compulsive Personality Disorder/<br>• Dependent Personality Disorder/<br>• Histrionic Personality Disorder/<br>• Hysteria/<br>• Paranoid Personality Disorder/<br>• Passive-Aggressive Personality Disorder/<br>• Schizoid Personality Disorder/<br>• Schizotypal Personality Disorder/
2	((personality or character*) adj3 disorder$).ti,ab,kw.	65164	
3	"axis II".ti,ab,kw.	1959	
4	("Complex trauma" or CPTSD or "complex post-traumatic stress disorder").ti,ab,kw.	535	
5	(Complex adj (needs or mental)).ti,ab,kw.	1929	
6	*Self-Injurious Behavior/	5465	
7	(Self-harm or self-injury).ti,ab,kw.	7484	
8	(emotion* adj2 (regulation or dysregulation or unstable or instability)).ti,ab,kw.	10588	
9	mood instability.ti,ab,kw.	254	
10	1 or 2 or 3 or 4 or 5 or 6 or 7 or 8 or 9	103674	2. Line two picks up the controlled indexing terms as free-text terms in the title (ti), abstract (ab) and author-generated keyword (kw). A proximity marker has been included (adj3) to identify personality within two words of the other terms in the search line. These free-text terms were verified against organisations such as MIND, for completeness. These search terms have

			been resented at a high-level for the purposes of scoping. The aim of this searching is not comprehensive but for a realistic sample of studies and data.<br><br>4. Search terms suggested by a member of the LEWG. Terms related to CPTSD which many of those diagnosed will have, and may provide useful models of care.<br><br>5. Suggested by a member of the LEWG. Services that don't used the personality disorder terminology often refer to themselves as "complex needs services" or "complex mental health"<br><br>6/7. Suggested by a member of the LEWG. Services for self-harm often see large numbers of people with personality disorder diagnoses.<br><br>8. Suggested by members of the LEWG.<br><br>10. combines the population-level search terms using the Boolean connect or, so that all terms represented are identified.
11	Community Health Services/	31050	11-18. These terms are for settings, including controlled indexing terms for relevant forms of health care provision and corresponding free-text terminology.<br><br>19. combines population and intervention terminology.
12	Community Mental Health Services/	18317	
13	((commun$ adj5 (mental health or model$1 or pathway$1 or program$ or evaluat$ or intervention$ or implement$)) or camhs or cmht$1).ti,ab,kw.	79584	
14	(community adj5 (agenc$ or care or center$ or centre$ or clinic$ or consultant$ or doctor$ or employee$ or expert$ or facilitator$ or healthcare or instructor$ or leader$ or manager$ or mentor$ or nurs$ or personnel$ or pharmacy or pharmacist$ or psychiatrist$ or psychologist$ or psychotherapist$ or specialist$ or skill$ or staff$ or team$ or therapist$ or tutor$ or visit$ or worker$ or group$ or independent or (peer$ adj3 support$) or survivor or outpatient$ or "out patient$")).ti,ab,kw.	96749	
15	(commun$ adj5 (service or hub$ or based or deliver$ or interact$ or led or maintenance or mediat$ or operated or provides or provider$ or run or setting$ or support or rehab$ or	205151	

	therap$ or service$ or treatment or management or assessment or assistance or care or day or week)).ti,ab,kw.		
16	(Independent sector or ((non institutional$ or noninstitution$) adj2 (sector$ or setting$))).ti,ab,kw.	367	
17	(network or outreach or ((specialist or day or whole) adj3 service)).ti,ab,kw.	351098	
18	((treatment* or (Dialectical behavior therapy or Dialectical behaviour therapy or DBT) or Psychotherapy* or specialist or psychiatry* or therapeutic or day or outreach or therap*) adj3 (Outpatient* or community or Service* or Center* or Centre* or Clinic*1 or Team* or program* or provider* or practice or setting* or care or community or unit* or hospital*)).ti,ab,kw.	244595	
19	11 or 12 or 13 or 14 or 15 or 16 or 17 or 18	851066	
20	Interview*.af.	373632	20-24. are the Wong broad filter for Qualitative studies (Wong et al. 2004)
21	Experience*.af.	1028333	
22	qualitative.tw.	212806	
23	Qualitative Research/	50164	
24	20 or 21 or 22 or 23	1444445	
25	randomized controlled trial.pt.	495635	25-45. are the Cochrane HSSS (Lefebvre, Manheimer, Glanville, 2011) and the SIGN observational search filter (Scottish Intercollegiate Guidelines Network, 2018) for studies reporting controlled trials and observational studies.
26	controlled clinical trial.pt.	93449	
27	(randomized or randomised).ab.	553632	
28	placebo.ab.	203251	
29	clinical trials as topic.sh.	189357	
30	randomly.ab.	322897	
31	trial.ti.	209094	
32	25 or 26 or 27 or 28 or 29 or 30 or 31	1289520	

33	Epidemiologic studies/	8156	
34	exp case control studies/	1037554	
35	Case control.tw.	120110	
36	(cohort adj (study or studies)).tw.	190004	
37	Cohort analy$.tw.	7484	
38	(Follow up adj (study or studies)).tw.	47969	
39	(observational adj (study or studies)).tw.	98982	
40	Longitudinal.tw.	232846	
41	Retrospective.tw.	497909	
42	Cross sectional.tw.	329612	
43	Cross-sectional studies/	311409	
44	33 or 34 or 35 or 36 or 37 or 38 or 39 or 40 or 41 or 42 or 43	2028189	
45	32 or 44	3197720	
46	"Surveys and Questionnaires"/	443562	46-47. represent a pragmatic search for studies reporting surveys.
47	survey$.tw.	612248	
48	exp clinical pathway/	6469	48-69. is the CADTH search filter for guidelines (CADTH, 2018).<br>70 combines the search filters.<br>71 includes a search for systematic reviews on this topic for the supplementary searches.<br>72 combines the population-level terms with the community-level intervention terms and the search filters.
49	exp clinical protocol/	163100	
50	exp consensus/	11712	
51	exp consensus development conference/	11685	
52	exp consensus development conferences as topic/	2772	
53	critical pathways/	6469	
54	exp guideline/	33000	
55	guidelines as topic/	38818	

56	exp practice guideline/	26125	
57	health planning guidelines/	4067	
58	(guideline or practice guideline or consensus development conference or consensus development conference, NIH).pt.	42151	
59	(position statement* or policy statement* or practice parameter* or best practice*).ti,ab,kf,kw.	31048	
60	(standards or guideline or guidelines).ti,kf,kw.	105440	
61	((practice or treatment* or clinical) adj guideline*).ab.	37832	
62	(CPG or CPGs).ti.	5569	
63	consensus*.ti,kf,kw.	24689	
64	consensus*.ab. /freq=2	23911	
65	((critical or clinical or practice) adj2 (path or paths or pathway or pathways or protocol*)).ti,ab,kf,kw.	19229	
66	recommendat*.ti,kf,kw.	39030	
67	(care adj2 (standard or path or paths or pathway or pathways or map or maps or plan or plans)).ti,ab,kf,kw.	55156	
68	(algorithm* adj2 (screening or examination or test or tested or testing or assessment* or diagnosis or diagnoses or diagnosed or diagnosing)).ti,ab,kf,kw.	7192	
69	(algorithm* adj2 (pharmacotherap* or chemotherap* or chemotreatment* or therap* or treatment* or intervention*)).ti,ab,kf,kw.	9314	
70	46 or 47 or 48 or 49 or 50 or 51 or 52 or 53 or 54 or 55 or 56 or 57 or 58 or 59 or 60 or 61 or 62 or 63 or 64 or 65 or 66 or 67 or 68 or 69	1422311	
71	(systematic adj3 review$).ti,ab,kw.	164344	
72	24 or 45 or 70 or 71	5190597	

73	10 and 19 and 72	3984	

2 – Methods

Detailed eligibility criteria:

- At least 50% of the sample should have a diagnosis of 'personality disorder' or related symptoms/diagnoses. Related symptoms include repeated self-harm or suicide attempts, complex trauma or complex PTSD, and emotional dysregulation or instability (We acknowledge this list is not comprehensive). The primary diagnosis or focus of treatment of the sample should be personality disorder or similar. Treatments that are primarily for other conditions are not included. This excludes qualitative studies of specialist treatment services for other diagnoses including substance misuse conditions.
- 90% of the sample should be over 16 years old or the sample should have mean sample age of 18 or over.
- Papers must report data related to care provided by community-based mental health services. Services can be a specialist 'personality disorder' service or a generic mental health/primary care/community service, as long as there is some reference to care for people with a diagnosis of personality disorder or related needs. For example, an eligible paper could describe initiatives within generic mental health/primary care service which focus on better meeting the needs of this group of people or describe the experiences of this group of people receiving generic services. Services for young people are excluded unless participants were transitioning to adult services. For example, qualitative data from people's experiences of moving from CAMHS to adult services will be included. Hospital services, such as day hospital treatments, were included provided participants still reside in the community through the course of treatment. Participants recruited from inpatient settings were included provided the treatment being evaluated was a community/outpatient treatment to which they have transitioned. Otherwise, inpatient and crisis services were excluded, as were forensic services and services specifically for offenders.
- Data from trials and other quantitative studies were included if the study offered a similar delivery/support to a community mental health team or specialist service, e.g. a good quality DBT treatment delivered by mental health practitioners embedded in a mental health service.
- Papers should present qualitative data and use recognised qualitative analysis methods (such as thematic analysis). Written data from questionnaires can be included if the data are analysed using a recognised qualitative method. Data from studies using Q methodology, a Delphi survey, or similar methods were excluded.

3 – Extended Results

1 - The long treatment journey

1.1 Skills learnt in treatment

Service users describe using skills learnt in treatment to achieve important clinical outcomes. Specifically, cognitive behavioural skills were identified as crucial for overcoming symptoms such as self-harm and impulsive behaviour. Dialectical Behavioural Therapy (DBT) was the most common treatment described as a practical way to learn new ways of thinking, and skills for managing feelings. Treatment also involved the acquisition of techniques such as mindfulness (where the service user focuses their attention in the present moment) and mentalization (where the service user focuses on their mental state to achieve objectivity over emotions for refection). At times, service users report understanding the skills but being unable to apply them successfully to their daily lives.

Over time, service users describe the skills learnt in treatment becoming 'second nature', improving incrementally in 'little steps.' The practice of skills in daily life made them become 'ingrained' and 'automatic' despite being difficult in the beginning. However, service users describe overwhelming emotions as a barrier to effective skill use. This left service users feeling demotivated to use skills in the future and feeling emotionally drained.

Service users describe 'personalisation' by focusing on skills that work best for them to control emotions and function in day-to-day interactions. Service users found personalisation of skills useful to overcome feelings of perfectionism which was found to be a barrier to effective skill use.

Emotional regulation was a key focus of skill development in treatment for service users. Negative emotions included anger, sadness, anxiety, fear, hopelessness and emptiness. Service users describe the challenges of managing overwhelming emotions leading to a loss of control. Specifically, being able to experience these emotions, allowing them to stay for an appropriate time, without engaging in activity such as self-harm or substance misuse. Loss of control was feared by service users, with safety associated with emotions being contained. Obtaining a feeling of control was an important component of recovery:

> "I think in terms of, like, recovery, in terms of being able to have a degree of self-control and being able to think ahead about the consequences of things so that rather than having a big blow up." (Shepherd et al., 2017)

Overwhelming emotions often related to individuals whom service users were emotionally attached. In cases where clients were unable to separate themselves from feelings in order to gain control, skill coaching was offered as treatment.

> "I think it's the opportunity to work out things and practise relationships with, come to a safe, safer place to practise ways of being… to practise getting angry, knowing that everyone is not going to leave you when you're angry. Seeing that you can make mistakes and that you, if you sit with it and work it through, that the world does not stop revolving." (Crawford et al., 2007)

1.2 Benefits and challenges of treatment

Service users often described treatment as 'life changing', giving them 'hope' for the future, and increased confidence and self-esteem. Treatment could help improve service users' understanding

of other people and be more mindful of the impact that their behaviour may have on them, which service users viewed as beneficial and improved their ability to relate to others. Having the skill set to manage symptoms enabled service users and helped to avoid negative coping mechanisms such as drug and alcohol use. In treatment, service users described learning skills to improve communication through the network of people encountered within services and within treatment, where skills could be refined and practiced. The ability to communicate effectively in turn strengthened relationships with friends and family, further adding to the benefits of treatment.

Treatment could provide service users with the space to reflect on their experiences, and to develop an awareness and understanding of the link between their thoughts, emotions, and behaviours. One key area was between their emotional state and the urge to self-harm. The benefits of treatment included insight obtained through art therapy and life story work. Service users described a ‘light bulb’ moment when identifying triggers for self-harm, which helped them better manage situations using coping strategies.

> “I don’t necessarily act on my thoughts anymore. My first reaction to something will be ‘I should self-harm’, but even though I’m not actually physically doing it, having my thoughts consumed by it is distressing.” (Ng et al., 2019b)

1.3 Change over time

Positive, stable, and ongoing support from clinicians contained distress over time. However, service users reported often feeling like they were not being supported satisfactorily in outpatient settings. The ‘healing’ effects of treatments developed over time, with service users describing how recovery fluctuated, oscillating between feeling in control, and being able to deal with problems, to feeling defeated and unable to cope with life.

> “Recovery was experienced as a series of achievements and setbacks, as SUs moved back and forth between these two poles of each recovery process. During this movement, they usually maintained an overall sense of moving forward, despite setbacks.” (Katsakou et al., 2017)

Service users describe trying again and again to use skills to overcome symptoms such as self-harm. Reflection on past behaviour and difficulties was described by service users as a key part of change and recovery:

> “I found that I was doing the same thing over and over again … unless you understand yourself I don’t think that … you can recover.” (Gillard et al., 2015)

Service users described gaining a new perspective from treatment which enabled a better understanding of themselves. This sense of understanding developed over time though their relationships with clinicians as well as other service users in group therapy.

1.4 Meaning

Service users describe how participation in meaningful activities, such as achievements outside treatment, contributed to a growth in confidence and a sense of purpose. Individual differences influenced what was considered meaningful, but examples of activities include parenting, homemaking, leisure activities, volunteering, and employment. Some service users attributed participation in such activities to be possible at least in part a result of treatment. The role of treatment was also described as supporting service users to gain meaning from past experiences.

2 - Client-clinician relationship

2.1 - Positive qualities for clinicians and the therapeutic relationship

A good therapeutic relationship was perceived to be at the centre of good care. However, in multiple papers, service users reported that forming and maintaining good relationships with their clinicians could be a significant problem. This could be aided by clinicians being warm, trustworthy, honest, open, accepting, non-judgemental, and interested in their job and in the service user as a person. Amongst these qualities, trust was often a priority and viewed as essential, and allowed service users to be open and engaged. For example, some service users reported only feeling comfortable revealing self-harm to their clinician if they trusted them with the information. Creating trust was a gradual process that took time. Being accepting, honest, and open, as well as setting clear boundaries without being punitive or judgmental, were all qualities in clinicians that helped establish trust (Fallon et al., 2003).

However, in multiple studies, service users reported that creating a trusting therapeutic relationship was not possible with some clinicians because they had poor rapport. Service users reported that clinicians should feel like good friends, who they felt understood them and were honest. Furthermore, many service users emphasised the importance of staff who had treated them like 'a person rather than ... a case number'. They wanted to be listened to and valued. In one study, a service user reported having a clinician who felt like they had a higher opinion of them than they had of themselves, and this relationship was viewed as especially beneficial therapeutically (Goldstein et al., 2015).

Loss of trust could also be precipitated by service users feeling like they had been let down by their clinicians, leaving them feeling alone with their difficulties. This left service users fearing 'abandonment' and 'rejection'. Similar feelings could come from sudden change in clinician or mental health service, or from suddenly being discharged from services. Service users advocated gradual change in their support teams, with careful and planned handovers.

> 'My psychiatrist has just left which has happened to me probably about a dozen times… it's quite a big part of my life, d'you not realise. These, from one appointment to the next is what's keeping me alive and they just don't say goodbye, they just don't even, it's ridiculous.' (Barr, Hodge, & Kirkcaldy, 2008)

Having a sense of safety and calm was important for creating a therapeutic environment. It helped service users feel more in control of themselves and their emotions and behaviour. For example, a feeling of safety was reported by service users as helpful for reducing self-harming. It was also important that the therapeutic environment and the clinicians were well suited to the needs of the service user and made them feel comfortable. In one study, some female service users reported only wanting to have female clinicians. Having clinical support during periods of significant distress was very important to service users in a number of studies. Although at times impossible for clinicians to achieve, service users in these studies were particularly appreciative of the support that they received during difficult times. They spoke positively of clinicians who they felt went 'above and beyond' the requirements of their role and expectations. Meanwhile, where service users had tried and failed to contact clinicians such times, it led to them feeling unimportant and devalued.

2.2 - Negative qualities

Problematic qualities for clinicians included being poorly or wrongly informed, and perpetuating stigmatising attitudes and therapeutic nihilism about Complex Emotional Needs (CEN). Meanwhile, in some studies, service users reported that their clinicians had been keen not to treat them or seemed disinterested, rushed through their appointments, or were dismissive, unsympathetic, or insensitive. Other negative characteristics included being overly strict, authoritarian, critical, superior, cold, or aloof. In several papers, service users said that these negative experiences were the rule for clinicians, and especially psychiatrists, with positive experiences being rarer. In one study, psychiatrists were described as 'quick, detached, uninvolved, and even sadistic'. While in another, a service user reported that:

> "…I felt like psychiatrists in general were like worthless and um didn't care about you and were only there to write you a prescription. And, so she was the first psychiatrist I ever saw who I thought gave a crap about me." (Goldstein et al.,2015)

> "I never felt that anyone really understood, everybody said they understood but then nothing at all happened. Everybody thinks that we are harming ourselves to get attention, but we are not, we are harming ourselves because life hurts so damned much. There was no respect for me as a human being. I had no value as a human whatsoever...and if I felt bad." (Perseius et al., 2006)

Other problematic characteristics included feeling like their clinician was judgmental or had behaved unethically or unprofessionally towards them. In one study, a service user described a doctor asking her if she was "stupid" after she was hospitalised due to overdose (Carrotte, Hartup, & Blanchard, 2019). Meanwhile, others reported that some clinicians had been overly strict and authoritarian, which they did not find therapeutic. At the extreme, clinicians were perceived as critical, superior, cold, or aloof:

> "I found it very, sort of, cold. There was no real warmth [...] It wasn't a warm environment.' 'I thought, "Why am I going to you? You're supposed to be making me feel better and you're not, you're just sitting there and you seem more depressed and fed up than I am." (Chatfield et al., 2013)

Not feeling understood or feeling like they were not being listened to was for service users in some studies not just irritating or disappointing but left them feeling that their clinician would be unable to help them. This was an experience that was reported for many types of clinician: General Practitioner (GP), care coordinator, psychiatrist, psychologist, amongst others. There was also reports, especially in relation to psychotherapy, that some service users felt damaged by their treatment: that the emotional demands of psychotherapy had proved too great or that therapists themselves had caused distress. In one study, a service user said:

> 'I just wanted them to admit that treatment was hard, maybe too hard for me. In a way, I felt battered deep inside me, but I only got treatment on the surface.' (Hummelen et al., 2007)

Feelings of rejection from services and clinicians were common across studies. In one study all the service users reported incidents with clinicians that made them feel rejected, which induced feelings of shame and anger (Goldstein, 2015). Some of the reported incidents were quite blatant, such as contacting private therapists who stated directly their unwillingness to work with anyone with a CEN diagnosis. While other experiences of rejection were subtler, such clinicians' general coldness or lack of concern about them.

2.3 - Relationship dynamics and structure

Across multiple studies, service users reported wanting to be challenged by their clinician, to be pushed to progress in their treatment, and to stop problematic coping strategies. Meanwhile, it was also important that clinicians understood the capabilities and limitations of the service user and did not push them too hard, which could be experienced as distressing or traumatic. As such, there was a careful balance that clinicians needed to achieve, while also being sure to adapt to the changing needs of service users over time. When this balance has not been achieved, so that the service user felt that the clinician was pushing too much or not enough, the treatment itself became less effective.

For many service users, the therapeutic relationship needed to feel like it was progressing through a collaborative and trusting discussion with their clinician(s). In the context of psychotherapy, service users in one study described this as having equality within the therapeutic relationship (Cunningham et al., 2004). Such relationships felt like friendships or partnerships in which the therapist and the service user were working towards the same, agreed upon goal. Such relationships gave service users a feeling of validation and empowerment.

In one study service users spoke about having ruptures in their therapeutic relationship with clinicians, and how those periods were handled was important (Goldstein et al., 2015). As discussed, service users wanted structure and clear boundaries, with some repercussions for when they broke the rules, and they wanted to be challenged by a therapist who understood them and who were attuned to their capabilities and limitations (Goldstein et al., 2015). However, it was also important that those repercussions were not overly punitive. Ruptures occurred out of conflict with the clinician, or when the service user had broken certain rules of the therapeutic relationship. Ruptures could be periods when the relationship was temporality changed or, more commonly, halted. But if clinicians managed overcoming these ruptures well, it helped service users feel secure, trusting, and confident or reassured in the clinician. Managing them well involved being open, accepting, non-judgmental, caring, and encouraging. Meanwhile, clinician neutrality, inactivity, judgment, or withholding of therapy following a rupture was viewed very negatively and lead some service users to 'call therapists' humanity into question' (Goldstein et al., 2015).

2.4 - Control: involvement in care and choosing who their clinicians are

While having a good therapeutic relationship was important, a recurring theme across papers was that service users often found it difficult to achieve because they had little or no control over who their allocated clinicians were. In some private mental health settings, service users had choice over their therapists was viewed as a major positive and improved the therapeutic value of the treatment that they received. Furthermore, service users often felt uninvolved during care programme approach meetings and care plan development and believed that greater collaboration was vital. Some service users reported that Care Plan Approach meetings often consisted of being told what staff had decided rather than consulting with the service users about what they believe might help and coming to a joint decision.

3) Mental Health Services

3.1 - Access to services

Across multiple studies, service users frequently mentioned their access to treatment. Whilst considering physical and financial resources were often outside of the remit of individual papers,

service users described difficulty getting transport to services, poor physical facilities and financial challenges in paying for treatment through private healthcare. One study referenced an app being used as part of therapy, which was helpful, but didn't always work (Helweg-Joergensen et al., 2019).

Service availability was another important issue, particularly in rural areas. Furthermore, many service users had experienced, or were at risk of, falling through service gaps. There were many reports of available services typically having long waiting lists, with waiting times in some cases over a year. Service users also noted a lack of specialist resources in many cases and could only access generalist services, which were perceived to be better than nothing, but significantly worse than specialist services.

In several studies, service users reported waiting a long time to be offered treatment at times. Once in treatment, they had a range of different experiences in terms of immediacy of access to support whilst under the care of services. Residential treatments generally had 24/7 support available, whilst day treatment often had nothing available outside of typical working hours. Flexible and easy access to professionals should service users need support in a crisis was valued, as was being provided with options for support. Similarly, some service users criticised the lack of options available for therapy and highlighted that there could be limited information about what was available to them and how they could advocate for themselves. DBT was sometimes the only choice available; although some conveyed a sense of being grateful to receive any treatment at all.

> "I'm still at the stage where if anyone offers me a service I'm just really grateful, so I'm not really going to criticise what it is." (Crawford, 2007)

3.2 Experiences of Mental Health Services

Overall, people had generally positive experiences of mental health services, and especially specialist services. Users reported services to be most helpful when staff were knowledgeable and warm, where service users were involved in their own care, such as their Care Plan Approach meetings, and where they had good access to high quality services that could offer treatment options well suited to their needs.

There have been many reports from service users of difficulties when receiving care from the mental health services. The severity of these challenges differed significantly between service users, with few experiencing a straightforward journey through the services. The first experience of the mental health services for many users is of their general practitioner (GP). Some service users also reported GP's could at times lack training in CEN, empathy, and understanding or willingness to work with them because they were perceived as 'difficult'. At its most extreme, some service users found these experiences to be traumatising. Many service users reported that their first contact with secondary mental health services was very confusing, and in many cases the confusion remained for some time. When they were first referred, service users would meet various mental health professionals, who at times could give highly variable explanations of their condition or how the services functioned. Some received no explanations at all concerning the roles of the individuals they were seeing or of the function of their contact with them. Correspondingly, there were reports of service users finding the assessment process difficult and daunting, and in some cases traumatic. Some said that was over-long and thorough, involving tests or questionnaires as well as interviews, and taking place over several weeks. Although some people appreciated the need for a comprehensive assessment process and felt that it engendered confidence in the staff and the service, many talked of the distress caused by focusing on painful past experiences and the difficult feelings this raised. It

made a considerable difference to service users if they felt that staff were there to answer questions and offer support.

Related to this, there were some reports from service users of staff, especially those in generalist rather than specialist services, lacking expertise or training in the condition, and offering little or incorrect information about the diagnosis and treatment options. The lack of knowledgeable staff resulted in service users feeling let down and rejected by services in some papers, especially if they had not found the typical treatment strategies beneficial. In multiple papers, service users reported experiences of being told to take new medications without any information of their function. This lack of information from staff led to service users seeking other resources, e.g. the internet; however, which could cause more harm than good. Some people also reported an overemphasis on the use of medication for treating CEN, rather than offering psychotherapy or other treatments.

Service users reported that transferring to the specialist service often led to a positive change in their treatment. Service users generally felt they received better support from specialist services than from generalist ones. In one study, service users reported that there was more involvement and choice in their treatment in a specialist team, which was important to them.

Movement through the services was often associated with a process of progress and relapse, which could occur very rapidly. Where service users felt that they could be open with the clinicians, they tended to view their treatment more positively and find it more productive. Where open discussion was lacking, service users felt their clinicians did not trust them and they tended to view their experience more negatively. For example, across papers, service users who felt involved in their Care Plan Approach decision-making tended to feel more positively about their treatment and that they were receiving the care they needed. In contrast, some service users reported finding Care Plan Approach meetings repetitive and said that nothing about their care seemed to change. While others reported experiencing their contact with the mental health services as a series of rushed outpatient appointments, or that they had been entirely excluded from important aspects of decision-making about their care. This could be compounded by a lack of consistency in their clinicians. For example, seeing doctors on rotation rather than the consultant.

Finally, there were many reports of service users finding the overall experience of using mental health services to be a 'blunt tool', which could not effectively respond to service users' needs particularly when things became difficult. Staff were also described as being focused on co-ordinating care, which was seen as useful, yet service users requested more psychological and emotional support. In this regard, what services provided was considered superficial and disproportionate with the levels of distress experienced.

There were reports from service users of a long process of determining which services and therapies they personally found most helpful. Some service users reported finding the greatest benefit from goal-directed, long-term, evidence-based therapies such as DBT and schema therapy, although not all service users were able to access such treatments. Furthermore, some service users noted an over-emphasis on DBT in the mental health services to the exclusion of other evidence-based treatments.

3.3 - Continuity of care

Consistency in care was important, especially around the clinicians who treated them. People often found change in clinicians or their mental health service challenging. However, there was also an acknowledgment that progression in their treatment could also require some difficult challenges at

times. As such, some service users said that change should be done over time to reduce the feeling of abandonment or rejection:

> "Because I've had that same service for 2 years it felt quite—I felt vulnerable [leaving]" (Rogers & Dunne, 2013)

Service users appreciated having long-term offers of therapy and having ongoing access to it when they were having difficult periods in their life. There was a sense that cut-off points for the ending of therapy could be quite arbitrary and could feel like they were putting pressure on service users to make improvements. Similarly, stability in certain elements of treatment was reassuring to service users: being able to work with the same professionals and having continued access to the same resources throughout their time under the care of services.

Transitions at the end of therapy needed to be handled carefully and could be particularly challenging for service users. Whilst for some ending therapy was a relief, others felt almost abandoned by their teams and it seemed to be common to be discharged from quite intensive support to very little or no support with no interim measures in place. According to one service user, "you're discharged from that service, then you're left high and dry" (Rogers & Dunne, 2013).

4 – Peer relationships

4.1 Family and friends

Service users discussed the involvement of family members such as spouses and parents in group therapy which facilitated a better understanding of CEN and improved communication and trust. Interpersonal relationships external to therapy were identified as an important form of support especially during difficult or distressing times. Service users described positive relationship as a virtuous cycle of recovery which could reinforce positive changes to thoughts and feelings and vice versa. Family and friends supported service users through encouragement, acknowledging change and mirroring the use of skills.

> "I can talk to my family more effectively about what's going on in my life, whereas before I was afraid to tell them what was happening…Most of it is communication. A lack of communication gives dark thoughts." (Cunningham, 2004)

Included articles highlight the importance of considering external sources of support during future service development specifically providing support for carers. Service users felt that carers needed to be offered more support to understand diagnosis and learn what to expect. Carer support groups provided education and information in addition to mutual support.

Service users describe how family environments, especially in childhood, can frame understanding and perception of experiences such as therapy. For example, experiences of abuse, neglect or violence was found to influence service users ability to engage with treatment due to a lack of validating relationships, and feeling silenced in sessions.

4.2 Peer support

In addition to relationships with friends, family and intimate partners, service users identified relations with clinicians and peers in therapy to be significant in supporting recovery. In some papers, service users highlighted the importance of relationships with others as part of the recovery

journey, for example hearing different ideas and opinions within group treatment. Unsupportive relationships were identified as a factor in the termination of therapy.

Service users identified shared experiences as the most powerful form of peer support within services, specifically encountering people in groups whom they could identify and share experiences. Over time, group therapy became a nurturing environment which enabled service users to feel 'normal' with peers becoming an 'adopted family.'

> "You realised that you weren't the only one feeling like that, there were other people in the world that felt the way that you did and being able to talk to them and hear their experiences of how they were dealing with it was helpful." (Crawford, 2007)

Reciprocal relationships provided a sense of belonging and inclusion for service users when interacting with others with similar experiences within group treatment. Service users provided support for one another specifically when managing symptoms such as self-harm. Informal conversations provided services users with the opportunity to share experiences as a form of support.

In some instances, friendships were extended outside of services. Peer-support networks involved meeting and supporting each other in and outside meetings, and sending texts or cards if others became unwell. This process of peer support was highly valued by some service users, where helping others increased an individual's self-esteem.

Service users at times found it difficult to balance wanting to help peers within groups, and being overpowering in terms of providing support. Service users also reported difficulty in balancing the giving and receiving of support, at times feeling like their own needs were not being met. This required time management within group treatment and the development of an awareness between the needs of others, and individual needs.

4.3 Group treatment

Service users describe how awareness of others during group treatment impacted self-harming behaviours, due to a sense of responsibility. Comparison to other members in the group facilitated understanding of different coping mechanisms, and identifying progress towards recovery. This awareness of others at times impeded therapeutic value of treatment with service users at times feeling inhibited by other group members sharing.

Service users described a strong sense of acceptance and belonging within CEN specialist services. Feelings of belonging from group treatment superseded the negative feelings which developed from highly emotional meetings. To overcome this, service users describe how 'ground rules' were set in therapeutic groups to create a 'culture of safety.' This involved service users talking about difficult emotions but setting boundaries to permissible behaviour. Services had different sanctions in place for breaches of ground rules, the most common rules surrounding self-harm and risky behaviour. At times, service users perceived rules to be punitive creating a barrier to disclosure of self-harm. Service users struggled with 'abstinence-based' approaches to self-harm as it discouraged honesty.

Service users appreciated an environment where members were encouraged to talk openly and reflect about behaviour and the emotional content in which it occurred, as this facilitated a culture of openness that was beneficial in treatment. However, on the other hand, some service users described being aware that the experiences they shared could trigger others in the group. Indeed, many service users described group treatment as an 'anxiety-provoking' experience, at times

describing groups as daunting, threatening and unsafe. Service users reported feeling anxious about feeling judged by clinicians or other group members and highlighted the importance of 'non-judgemental ethos' within groups. This anxiety was also shared with established members of groups when new members joined and was found to limit service users' ability to use groups as a resource when not managed effectively, expressing a preference for individual therapy. Anxiety during group therapy was associated with dropout from treatment, and clinicians had an important role in monitoring these feelings to explore, validate and target them when required.

Dynamics between group members could create feelings of exclusion and marginalisation for some service users. Service users also reported having to manage dynamics within groups to have an opportunity to participate in group discussion. Time constrains and size of group were described as challenges to ensuring equal time to discuss experiences across all service users. Service users experienced the collective concern within groups to be supportive. However, hearing experiences of other group members could be detrimental to service user wellbeing in the short-term.

> "Because it can, because you‘re hearing other people‘s stories as well, and if you‘re not used to hearing that kind of information it can be quite alarming and it‘s like, oh I don‘t want to hear this, or I don‘t feel comfortable with this, so people have to be prepared you know, there are worse things that‘ve gone on in other people‘s lives." (Barr, Hodge, & Kirkcaldy, 2008)

5 - Engaging with treatment: psychological barriers and facilitators

5.1 - Barriers to treatment

A prominent difficulty with engaging with treatment was overwhelming emotions experienced by individuals, generally linked to their symptoms. These were often seen as impeding service users from practising coping skills learnt in treatment.

Service users often believed that the emotional demands of therapy sessions had a general negative impact on them. In the hours after therapy sessions it was common to experience feelings of distress and vulnerability as a result of difficult emotions being exacerbated and traumatic memories being triggered.

> "When I started day treatment I felt so vulnerable, almost skinless. So many repulsive feelings were aroused during DT, but I just felt left alone with those feelings." (Hummelen et al, 2007)

Therapies taking place in group settings could be daunting as service users had to listen to the experiences of others and therefore absorb other people's emotions and to hear about challenges that reminded them of their own difficult experiences.

Service users also experienced challenges related to their learning. The idea of learning new skills was difficult for some, especially when they weren't sure that these skills could work for them. It could also be challenging to understand the material that was being taught.

5.2 - Facilitators of treatment

Both service users and staff leading treatments found it beneficial if service users were able to take ownership over their treatment. Key elements of this were service users being self-motivated to

seek support for making a change and to engage with therapy, and being able to identify and focus on the skills that they personally thought would be helpful.

> "I have got a responsibility, it's me that has to change things with support from my therapist" (Perseus et al, 2003)

Similarly, taking personal context into account was believed to be helpful, particularly in terms of attachments and trauma that may have happened in the past. Service users sometimes identified reframing or making sense of their past as a marker of success in treatment.

Positive interactions with professionals could also be important; service users valued feeling understood and cared for by staff and other members of group treatments. Clear communication from staff throughout the treatment pathway about what treatment entailed was important, especially to help service users manage their expectations of what would come of it.

5.3 - Recovery

Different service users had different ideas of what recovery meant to them, as they continued to cope with the ongoing challenges of managing their mental health after treatment. Across papers, it was often accepted that borderline personality disorder (BPD) symptoms wouldn't ever disappear; rather, recovery was seen as a positive change where individuals still had to deal with their illness but had more control over their thoughts and emotions, or had reduced the negative impact BPD had on their life.

In some papers, service users felt quite negative about the concept of recovery, with one study highlighting that the concept of recovery and change can be quite daunting for people with BPD. Service users also experienced relapses and "ups and downs", which left them feeling discouraged about their potential to recover.

> "Yesterday was relatively ok, today is ok so far. But before, consistently, I had a period where I couldn't actually leave the house and I was very dissatisfied and self-hating… So it's difficult to actually trust the times when I am feeling alright." (Katsakou, 2012)

6 – Components of treatment

6.1 - Individualised treatment

Service users emphasised the importance of individualised care rather than having a 'one size fits all' approach. Service users felt that clinicians sometimes focused too much on their diagnosis or relied too heavy on delivering the components of certain formal therapies, such as Dialectical Behavioural Therapy (DBT). Similar issues could arise when service users felt like they were being treated as 'just another' number or patient. There were a number of reports from service users who felt that they had been de-individualised, and were being treated as merely 'a number on a caseload' with little meaning or value to care coordinators. This was an issue at times, for example, when care coordinators seemed disinterested, demotivated, or services felt over-stretched.

It was also important that individualised approaches were well tailored to the service user. Some found that there was a clash between their personal aspirations and the focus their treatment was given by clinicians. They felt that therapy did not address the problems they were struggling with and focused almost exclusively on specific topics, i.e. self-harming or relationships, leaving service

users frustrated when they could not address other issues that were either equally or more important to them, such as their past traumatic experiences.

6.2 - Structured Approach

Service users general spoke positively of having a structured approach to treatment. They especially valued therapies that offered a clear structure and a treatment rationale that was discussed with them. Reflecting this, service users valued rules for therapy that were straightforward and clear, without being punitive or judgmental. While this applied in many contexts, some clear examples came from group therapy and from rules around self-harming. One service user reported abstaining from self-injury ever since clinicians had introduced clear rules as part of their therapy and said that they would have continued to cut if the team had not introduced them. But they also said that a context of "compassion and understanding" along with continued access to clinician warmth and comfort was crucial to withstand these rules.

> "[The structure] certainly has an effect on the relationship. Because I think that makes a difference, that makes me more positive." (van Veen et al., 2019)

In some papers, service users talked about the sense of routine and structure that having regularly scheduled sessions brings to their lives. Furthermore, ensuring therapy has appropriate structure and was well-defined enabled service users to focus on and explore specific challenges better, which helped provide continuity and consistency in care. For example, in groups, allotting time to all participants helped ensure everyone had the opportunity to speak. Service users receiving group therapy often spoke negatively about 'unstructured' group time as it meant that a few service users often dominated discussion. However, it is also important to allow some flexibility in the rules to allow the needs of individuals to be met. Moreover, having rules that were too strict or rigorously enforced led to distress for some service users. For example, rules that prevent open discussion of certain feelings and behaviours were experienced as frustrating, dehumanising, and distressing by service users, such as in the case of self-harming.

6.3 - Self-Harm

One area where a structured approach was important was self-harm. In general, across papers service users spoke about wanting to control or stop self-harming:

> ''Stopping self-harming was one of my goals… It got to the point where it was a thing as an addiction. The moment I felt even the slightest bit of stress I was cutting, so even I knew it escalated and I needed…I don't think I necessarily wanted to stop but I wanted to control it, ultimately it stopped'' (Katsakou et al. 2012)

Service users spoke positively about structured approaches to supporting them to reduce or stop self-harming. It was important that a good balance between being overly punitive and permissive was achieved. Approaches that were overly punitive to be distressing or stigmatising. These strategies could intensify people's negative experiences of being excluded or judged and could increase people's inclination to self-harm. Moreover, overly strict approaches were perceived as coercive and encouraged service users not to be open with their clinicians. Meanwhile, overly permissive attitudes were also viewed negatively because they did not provide service users with motivation to quit:

> ‘Exclusion or discharge is too punitive I guess… [But] this [group] is the other side of the coin… You know people's self-destruction has no consequences, it's gone, it's gone completely to the other extreme.’ (Barr, Hodge, & Kirkcaldy, 2008)

Instead, approaches that helped people develop alternative coping strategies and that felt encouraging were preferred. The stated goal did not always need to be to eliminate self-harming, but instead to explore alternative ways to cope with intense emotions or reduce their need for self-harming.

> ‘‘It wasn’t until I did a lot of dealing with the issues that were underneath it that it was possible for me to stop.’’ (Long, Manktelow, & Tracey 2016)

Furthermore, having a clear structure that encouraged service users in this way to reduce their self-harming were often perceived as a sign of the clinician caring about the service user’s well-being, and feeling cared about had substantial therapeutic value. Although sometimes being encouraged to stop self-harming could be perceived as difficult or annoying. Furthermore, an individualised approach, that suits the needs and difficulties faced by the service user, is important in many contexts. In studies of group therapy, if the self-harming behaviour of members was not being sufficiently challenged, some service users felt it allowed for an over-emphasis on negative ways of coping and perhaps to members using self-harm as a means of gaining or keeping the attention of the group.

In some papers, self-harming was generally seen by service users as a coping strategy for dealing with unbearable emotional pain and distress and thus represented a life-saving, rather than life-ending, strategy. However, service users reported that clinicians often did not understand this. This was especially true outside of mental health services, where some clinicians saw self-harming as destructive or about ‘enjoying pain’. For example, one participant reported being asked by a doctor whether they wanted anaesthetic during suturing, commenting, ‘Well, you obviously did this to yourself, so you like pain’. Service users with significant histories of self-harming especially described experiencing discrimination. For some, the scars from self-harming had become a permanent part of their identity and had led to stigma. One service user described how they gave her the identity of a ‘sick person’.

Moreover, within mental health services, some service users spoke about finding it very frustrating when service providers seemed not to appreciate the intensity of their emotions, and suggested coping strategies that were largely ineffective, like having a bath. Service users knew using self-harming as a coping strategy caused them injury or was damaging, but in the absence of more constructive strategies or support, they felt that they had little choice.

6.4 - Medications

Service users sometimes felt like they were ‘guinea pigs’ who were trialled on numerous medications because staff did not know how to treat them.

> “I just think when you first come into service that they experiment on you . . . over the course of years they’ve experimented with lots of different drugs. I’ve felt like they didn’t understand, and they just like piled me with any sort of medication.” (Rogers & Acton, 2012)

They also felt there was an overemphasis on the use of medication for treating BPD, rather than offering other types of treatment, despite the lack of evidence supporting its efficacy. Furthermore,

service users repeatedly identified that they were poorly informed of the rationale for their treatment, particularly when they were being treated in an inpatient setting.

> “They don’t say why that particular [drug] or anything – they just give them to you. She said ‘you either swallow it, or we’ll give you an injection’ so no choice.” (Rogers & Acton, 2012)

This experience changed when moved to the specialist service, where they were given the choice of including medication as part of their treatment. Involvement in decisions around their treatment allowed service users the power to decide on their own recovery pathway, which varied between individuals. However, choosing to include medication as part of their recovery meant service users had to learn to manage the side effects associated with the medication.

7 – Diagnosis

Service user views of diagnosis were mixed. Two key themes were the importance of diagnosis for accessing services and how service users themselves understand the diagnosis.

7.1 Self-understanding

Negative aspects of receiving the diagnosis included stigma or being stereotyped, including from clinicians and society-at-large, a sense in which the diagnosis served only to medicalise their distress, and for some people, being excluded from services. There were many reports of service users feeling apprehension about receiving a CEN diagnosis due to potentially negative attitudes from others. Consistent with this, many service users thought that the CEN diagnosis was a derogatory term that they strongly disliked. Moreover, many service users reported that receiving the diagnosis negatively affected their clinicians’ perceptions and the treatment they received:

> “I feel like once you get a diagnosis of BPD they sort of act like you are kind of beyond their...bother. Like they don’t especially want to do anything because you are not going to be easy.”

For some, receiving a CEN diagnosis meant that they were no longer seen as unwell or distressed, but instead as ‘difficult’. Some symptoms that they experienced, such as those of psychosis, were no longer viewed as genuine by staff. Problematic attitudes amongst clinicians were particularly prevalent in generalist services and primary care but were present in secondary mental health services too. Users of these services often reported that some clinicians lacked training in CEN, empathy, and understanding. One service user reported being told by a psychiatrist that:

> “Oh God, no, I don’t treat borderlines, they’re so difficult, I run from them… I run from them, they’re so difficult, they make my life crazy.” (Goldstein, 2015)

For some, the contested and uncertain nature of the CEN diagnosis made it more difficult to feel in control of their condition because there were so many myths, misinformation, and derogatory attitudes, including amongst their clinicians. Such views include that CEN is untreatable, that self-harming and other behaviours are manipulations to gain attention, and that service users with CEN are liars, attention-seeking, unreasonable or difficult, manipulative, and taking resources from other patients. Some service users reported that the stigma and myths held by clinicians, and they way that their treatment changed following receiving the diagnosis, compounded their feelings of isolation, marginalisation, or abandonment or rejection. Furthermore, for some, receiving the diagnosis left them feeling like they were being pathologized for having a disorder that they

developed due to the abuse that they had experienced, causing them to feel like victims all over again.

7.2 Access to treatment and communication of diagnosis

Positive aspects of receiving the diagnosis included that it allows better access to appropriate treatment and, in some reports, helped people improve their self-understanding. In many papers, receiving a diagnosis was a turning point for service users in their recovery. It could help service users conceptualise their own experiences and emotional intensity. This was especially true if the diagnosis seemed to fit their experiences, it was contextualised with helpful information about the condition and treatment options, and if it meant that services users could access therapy that they were previously excluded from. In such situations, the diagnosis could offer a sense of validation and relief.

How service users were told about their diagnosis was important for how they felt about it subsequently. Some said that receiving the diagnosis was fraught with confusion and misinformation, and it was often a long road to diagnosis. Others reported being given their diagnosis in an insensitive manner with little opportunity for discussion. Being given the diagnosis by a clinician who understood the condition, who had time for discussion, and who was optimistic about the effectiveness of treatment and the likelihood of recovery improved perceptions of the diagnosis.

However, service users who were given the diagnosis not in this way, or in a way that they perceived as problematic or unhelpful, tended to view it more negatively. This includes if the diagnosis had been poorly communicated. Some reported that they had only discovered their diagnosis by accident, for example, on the back of a Disability Living Allowance form or on a hospital discharge certificate. Another problem was when clinicians had given them misinformation or frustrating and confusing messages about their condition. One example of this was service users being given the diagnosis because nothing else fitted. This left service users feeling like their clinicians did not really understand what their condition was.

Attempts by clinicians to avoid or sidestep the CEN or ‘personality disorder’ diagnosis were seen as counter-therapeutic by some because they were inadvertently indicating their negative attitudes about the condition and, possibly, invalidating service users hopes and understanding of themselves. However, this does not mean that every service user found the diagnosis useful. Instead, there was a broad view that clinicians should assess the meaning and utility of a CEN diagnosis for the service user and take care not to assume they will find relief in receiving the diagnosis or that the diagnosis is harmful.

References

Barnicot K, Couldrey L, Sandhu S, Priebe S. Overcoming barriers to skills training in borderline personality disorder: A qualitative interview study. PLoS ONE. 2015 Oct 14; 10(10): e0140635

Barr W, Hodge S, Kirkcaldy A. Evaluation of the day services run by agency partnerships and therapeutic community service north. Liverpool: Health and Community Care Research Unit, University of Liverpool, 2008. 166p.

Bradbury L. A qualitative exploration of the relationship between those with a diagnosis of Borderline Personality Disorder and their care coordinators: a service user perspective. Doctoral Thesis, University of Surrey. 2016. Available from: https://core.ac.uk/display/76988193

Carrotte E, Hartup M, Blanchard M. "It's very hard for me to say anything positive": A qualitative investigation into borderline personality disorder treatment experiences in the Australian context. Aust Psychol. 2019 May 02; 54(6): 526-535.

Castillo H, Ramon S, Morant N. A recovery journey for people with personality disorder. Int J Soc Psychiatry. 2013 Mar 12; 59(3): 264-273.

Chatfield J. Investigating client dropout from psychotherapeutic treatments for personality disorder. Doctoral Thesis, Canterbury Christ Church University. 2013. Available from: https://repository.canterbury.ac.uk/download/73cf7d68af8225635cfd5266492b31f30302aa707471a0848f023c60d16e2be2/1712935/Jon_Chatfield_MRP_2013.pdf

Ciclitira K, Starr F, Payne N, Clarke L, Marzano. A sanctuary of tranquillity in a ruptured world: Evaluating longterm counselling at a women's community health centre. Fem Psychol. 2017 Jan 25; 27(4): 530–552.

Clarke JM, Waring J. The transformative role of interaction rituals within therapeutic communities. Sociology of Health & Illness. 2018 Jun 29; 40(8): 1277–1293.

Clarke JM. The case for "fluid" hierarchies in therapeutic communities. Ther Communities. 2017; 38(4): 207-216.

Cooper C, Dawson S, Peters J, Varley-Campbell J, Cockcroft E, Hendon J, et al. Revisiting the need for a literature search narrative: A brief methodological note. Res Synth Methods. 2018;9(3):361-5.

Crawford M, Rutter D, Price K, Weaver T, Josson M, Tyrer P, et al. Learning the lessons: a multimethod evaluation of dedicated community-based services for people with personality disorder. London: National Coordinating Centre for NHS Service Delivery and Organisation; 2007. 381 p.

Cunningham K, Wolbert R, Lillie B. It's About Me Solving My Problems: Clients' Assessments of Dialectical Behavior Therapy. Cognitive and Behavioral Practice. 2004; 11(2): 248-256.

Donald F, Duff C, Broadbear J, Rao S, Lawrence K. Consumer perspectives on personal recovery and borderline personality disorder. The Journal of Mental Health Training, Education and Practice. 2017 Nov 06; 12(6): 350-359.

Falconer CJ, Cutting P, Davies EB, Hollis C, Stallard P, Moran P. Adjunctive avatar therapy for mentalization-based treatment of borderline personality disorder: a mixed-methods feasibility study. Evidence Based Mental Health. 2017 Oct 22; 20(4): 123-127.

Fallon P. Travelling through the system: the lived experience of people with borderline personality disorder in contact with psychiatric services. J Psychiatr Ment Health Nurs. 2003 Jul 21; 10(4): 393-401.

Flynn S, Raphael J, Graney J, Nyathi T, Williams A, Kapur N, et al. The personality disorder patient pathway: Service user and clinical perspectives. Personality and Mental Health. 2019 May 20; 13(3): : 134–143.

Folmo EJ, Karterud SW, Kongerslev MT, Kvarstein EH, Stänicke E. Battles of the Comfort Zone: Modelling Therapeutic Strategy, Alliance, and Epistemic Trust—A Qualitative Study of Mentalization-Based Therapy for Borderline Personality Disorder. J Contemp Psychother. 2019; 49(3): 141-151.

Gillard S, Turner K, Neffgen M. Understanding recovery in the context of lived experience of personality disorders: a collaborative, qualitative research study. BMC Psychiatry. 2015 Jul 31; 15(183): 1-13.

Gillard S, White R, Miller S, Turner K. Open access support groups for people experiencing personality disorders: Do group members' experiences reflect the theoretical foundations of the SUN project? Psychology and Psychotherapy: Theory, Research and Practice. 2015 Mar 01; 88(1): 87–104.

Goldstein SE. A narrative study of the relationships between women diagnosed with borderline personality disorder and their therapists. Doctoral Thesis, Fielding Graduate University. 2015. Available from: (FIND WEBSITE FOR ACCESS)

Haeyan S, Kleijberg M, Hinz, L. Art therapy for patients diagnosed with personality disorders cluster B/C: A thematic analysis of emotion regulation from patient and art therapist perspectives. International Journal of Art Therapy. 2018; 23(4): 156-168.

Helweg-Joergensen S, Schmidt T, Lichtenstein MB, Pederson SS. Using a Mobile Diary App in the Treatment of Borderline Personality Disorder: Mixed Methods Feasibility Study. JMIR Form Res. 2019 Sep 30; 3(3): e12852.

Hodgetts A, Wright J, Gough A. Clients with borderline personality disorder: Exploring their experiences of dialectical behaviour therapy. Counselling and Psychotherapy Research. 2007 Aug 10; 7(3): 172-177.

Hummelen B, Wilberg T, Karterud S. Interviews of Female Patients with Borderline Personality Disorder Who Dropped Out Of Group Psychotherapy. International Journal of Group Psychotherapy. 2007; 57(1): 67-92.

Katsakou C, Marougka S, Barnicot K, Savill M, White H, Lockwood K, et al. Recovery in Borderline Personality Disorder (BPD): A Qualitative Study of Service Users' Perspectives. PLoS ONE. 2012 May 17; 7(5): e36517.

Katsakou C, Pistrang N, Barnicot K, White H, Priebe S. Processes of recovery through routine or specialist treatment for borderline personality disorder (BPD): a qualitative study. J Ment Health. 2017; 28(6). Available from:

https://www.tandfonline.com/doi/full/10.1080/09638237.2017.1340631 - Conflicting Publication dates – Latest pub. Is 2019.

Larivière N, Couture E, Blackburn C, Carbonneau M, Lacombe C, Schinck S, et al. Recovery, as Experienced by Women with Borderline Personality Disorder. The Psychiatric Quarterly. 2015 Mar 04; 86(4): 555-568.

Lefebvre C, Manheimer E, Glanville J. Chapter 6: Searching for studies. In: Higgins JPT, Green S. (eds.) Cochrane Handbook for Systematic Reviews of Interventions Version 5.1.0 (updated March 2011). The Cochrane Collaboration; 2011 [Accessed 7th December 2017]. Available from: http://handbook.cochrane.org/

Leung M, Chow C, Ip PP, Yip SP. Self-harm attempters' perception of community services and its implication on service provision. J Nurs Home Res Sci. 2019 Jan 10; 6(1): 50-57.

Lohamn MC, Whiteman KL, Yeomans FE, Cherico SA, Christ WR. Qualitative Analysis of Resources and Barriers for Borderline Personality Disorder in the U.S. Psychiatric Services. 2017 Feb 01; 68(2): 167-172.

Lonargain DO, Hodge S, Line R. Service User Experiences of MentalisationBased Treatment for Borderline Personality Disorder. Mental Health Review Journal. 2017 Mar 13; 22(1): 16-27.

Long M, Manktelow R, Tracey A. "Knowing that I'm not alone": client perspectives on counselling for self-injury. J Ment Health. 2016; 25(1): 41-46.

McSherry P, O'Connor C, Hevey D, Gibbons P. Service user experience of adapted dialectical behaviour therapy in a community adult mental health setting. J Ment Health. 2012; 21(6): 539-547.

Morant N, King J. A multi-perspective evaluation of a specialist outpatient service for people with personality disorders. J Forens Psychiatry Psychol. 2003; 14(1): 44-66.

Morris C, Smith I, Alwin N. Is contact with adult mental health services helpful for individuals with a diagnosable BPD? A study of service users views in the UK. J Ment Health. 2014 Sep 15; 23(5): 251-255.

Mortimer-Jones S, Morrison P, Munib A, Paolucci F, Neale S, Hellwell A, et al. Staff and client perspectives of the Open Borders programme for people with borderline personality disorder. Int J Ment Health Nurs. 2019 May 12; 28(4): 971–979.

Naismith I, Kerr S, Mwale A, Feigenbaum J. A thematic analysis of compassion-focused imagery for people with personality disorder: Inhibitors, facilitators and clinical recommendations. Clinical Psychologist. 2019 Jun 04; 23(3): 213–224

Ng FYY, Carter PE, Bourke ME, Grenyer BFS. What Do Individuals With Borderline Personality Disorder Want From Treatment? A Study of Self-generated Treatment and Recovery Goals. J Psychiatr Pract. 2019; 25(2): 148-155.

Ng FYY, Townsend ML, Miller CE, Jewell M, Grenyer BFS. The lived experience of recovery in borderline personality disorder: a qualitative study. Borderline Personal Disord Emot Dysregul. 2019 May 22; 6(10): 1-9.

Perseius K, Ekdahl S, Åsberg M, Samuelsson M. To Tame a Volcano: Patients with Borderline Personality Disorder and Their Perceptions of Suffering. Arch Psychiatr Nurs. 2005; 19(4): 160-168.

Perseius K, Ojehagen A, Ekdahl S, Åsberg M, Samuelsson M. Treatment of Suicidal and Deliberate SelfHarming Patients With Borderline Personality Disorder Using Dialectical Behavioral Therapy: The Patients' and the Therapists' Perceptions. Arch Psychiatr Nurs. 2003; 17(5): 218-227.

Rogers B, Acton T. 'I think we're all guinea pigs really': a qualitative study of medication and borderline personality disorder. J Psychiatr Ment Health Nurs. 2012; 19(4): 341–347.

Rogers B, Dunne E. A qualitative study on the use of the care programme approach with individuals with borderline personality disorder. J Psychosoc Nurs Ment Health Serv. 2013; 51(10): 38-45.

Scottish Intercollegiate Guidelines Network. SIGN: Search Filters 2018 [Accessed 12th February 2019]. Available from: https://www.sign.ac.uk/search-filters.html

Sheperd A, Sanders C, Shaw J. Seeking to understand lived experiences of personal recovery in personality disorder in community and forensic settings – a qualitative methods investigation. BMC Psychiatry. 2017 Aug 01; 17(282): 1-10

Smith D. Experiences of Dialectical Behavioural Therapy by Adults Diagnosed with Borderline Personality Disorder. Doctoral Thesis, University of Liverpool. 2013. Available from: https://livrepository.liverpool.ac.uk/13733/2/SmithDon_Jun2013_13733_(abridged).pdf

Stalker K, Ferguson I, Barclay A. 'It is a horrible term for someone': service user and provider perspectives on 'personality disorder'. Disability & Society. 2010 Oct 01; 20(4): 359-373.

The Canadian Agency for Drugs and Technologies in Health (CADTH). Strings Attached: CADTH's Database Search Filters 2018 [Accessed 12th February 2019]. Available from: https://www.cadth.ca/resources/finding-evidence/strings-attached-cadths-database-search-filters#guide

van Veen M, Peters A, Mulder N, van Meijel B, Koekkoek B. A Qualitative Study of the Working Alliance between Patient and Community Mental Health Nurse during Interpersonal Community Psychiatric Treatment. Issues in Mental Health Nursing. 2019; 41(3): 211-220.

Veysey S. People with a borderline personality disorder diagnosis describe discriminatory experiences. Kotuitui: New Zealand Journal of Social Sciences Online. 2013; 9(1): 20-35. Conflicting Publication dates – Latest pub. Is 2019.

Walker T. 'Seeing beyond the battled body' An insight into selfhood and identity from women's accounts who self-harm with a diagnosis of borderline personality disorder. Counselling and Psychotherapy Research. 2009 May 14; 9(2): 122-128.

Wong SS, Wilczynski NL, Haynes RB. Developing optimal search strategies for detecting clinically relevant qualitative studies in MEDLINE. Stud Health Technol Inform. 2004;107(Pt 1):311-6.
